# FoxO3 suppresses Myc-driven lymphomagenesis

**DOI:** 10.1038/cddis.2015.396

**Published:** 2016-01-14

**Authors:** C J Vandenberg, N Motoyama, S Cory

**Affiliations:** 1 Molecular Genetics of Cancer Division, The Walter and Eliza Hall Institute of Medical Research, Melbourne, Victoria, Australia; 2 Department of Medical Biology, University of Melbourne, Melbourne, Victoria, Australia; 3 Department of Human Nutrition, Sugiyama Jogakuen University School of Life Studies, Chikusa-ku, Nagoya, Aichi, Japan

## Abstract

This study demonstrates, for the first time, that loss of a single forkhead box class O (FoxO) transcription factor, can promote lymphomagenesis. Using two different mouse models, we show that FoxO3 has a significant tumour-suppressor function in the context of Myc-driven lymphomagenesis. Loss of FoxO3 significantly accelerated myeloid tumorigenesis in vavP-*MYC10* transgenic mice and B lymphomagenesis in Eμ-*myc* transgenic mice. Tumour analysis indicated that the selective pressure for mutation of the p53 pathway during Eμ-*myc* lymphomagenesis was not altered. Frank tumours were preceded by elevated macrophage numbers in *FoxO3*^−/−^ vavP-*MYC10* mice but, surprisingly, pre-B-cell numbers were relatively normal in healthy young *FoxO3*^−/−^Eμ-*myc* mice. *In vitro* assays revealed enhanced survival capacity of Myc-driven cells lacking FoxO3, but no change in cell cycling was detected. The loss of FoxO3 may also be affecting other tumour-suppressive functions for which FoxO1/4 cannot fully compensate.

The evolutionarily conserved forkhead box class O (FoxO) transcription factors have important roles in cellular metabolism, stress tolerance and probably lifespan.^1, 2^ They are key regulators of cell proliferation and survival, a large number of their transcriptional targets being involved in apoptosis,^3, 4, 5^ cell cycle arrest,^6, 7^ DNA repair, oxidative stress resistance and metabolic processes.^8, 9^

Regulation of FoxOs is complex, involving acetylation and ubiquitination as well as phosphorylation (reviewed in Eijkelenboom and Burgering^10^). Under normal conditions of growth factor signalling, FoxO transcription factors are inactivated through phosphorylation by activated AKT. For FoxO3 this occurs at three conserved residues (Thr-32, Ser-253 and Ser-315), resulting in the export of FoxO3 from the nucleus into the cytoplasm^11^ and proteasomal degradation.^12^ During conditions of oxidative stress, nuclear translocation and activation of FoxOs occurs through Jun N-terminal kinase phosphorylation, which overrides growth factor signalling.^13^

The FoxOs are highly related and to some degree can act redundantly as they bind to the same consensus sequence.^14^ Of the four mammalian FoxO family members, FoxO1, FoxO3 and FoxO4 are widely expressed,^14^ whereas FoxO6 is mainly expressed in adult brain tissue.^15^ In haemopoietic tissues, FoxO1 and FoxO3 are the major FoxOs, with FoxO1 levels highest in lymphoid cells and FoxO3 levels highest in myeloid cells.^16^

Although *FoxO4*^−/−^ mice do not have an obvious phenotype,^17^ *FoxO6*^−/−^ mice show impaired memory consolidation^18^ and deletion of *FoxO1* results in defects in embryonic vascular development, resulting in death at embryonic day 10.5.^17, 19^ FoxO3-deficient mice display early onset female infertility because of depletion of ovarian follicles caused by widespread follicular activation.^17, 20^ Aged *FoxO3*^−/−^ mice were found to have reduced numbers of haemopoietic stem cells (HSCs),^21^ implying that FoxO3 contributes to the maintenance of the HSC pool during aging by reducing oxidative stress.^22^ FoxO3 primes HSCs for metabolic stress (starvation)-induced autophagy, which is necessary for HSC survival.^23^

Activation of AKT, and thus inactivation of FoxOs, is frequently observed in a range of cancers,^24^ raising the possibility that FoxO proteins may serve as tumour suppressors. An early study found that loss of individual FoxOs did not cause cancer predisposition, except for loss of FoxO1.^17, 20^ When FoxO1 was deleted using Mx-Cre (at 4 weeks of age), mice developed systemic hemangiomas, but with long latency and low frequency.^25^ However, compound somatic deletion of three FoxOs, FoxO1/3/4, resulted in thymic lymphomagenesis.^25^ Furthermore, a dominant-negative FoxO construct, which expresses the conserved DNA-binding domain of FoxO4 but lacks the transactivation domain and thus inhibits the activity of all FoxOs, greatly accelerated the onset of Eμ-*myc* lymphomas.^26^ This effect is due, at least in part, to FoxO upregulation of p19^ARF^ expression, which promotes p53-dependent apoptosis. In fact, the dominant-negative FoxO Eμ-*myc* tumours arose as rapidly as those on a p53^+/−^ background.^26^ FoxO3 and MYC compete for binding to some promoters and appear to antagonise each other's activity.^27, 28, 29^ FoxO3 directly regulates expression of the MYC inhibitor MXI1, whereas other Max-interacting protein (MXD) family members are regulated indirectly.^30^

To determine whether loss of a single FoxO can cooperate in Myc-driven tumorigenesis, we crossed *FoxO3*^−/−^ mice^21^ with vavP-*MYC*10 mice, which express the transgene in all haemopoietic lineages and primarily develop myeloid tumours with some T lymphomas,^31, 32^ and with Eμ-*myc* mice, which constitutively express Myc in B lymphoid cells and are susceptible to pre-B and B lymphomas.^33, 34^

## Results

### Loss of FoxO3 accelerates Myc-driven tumorigenesis

**Figure 1:**
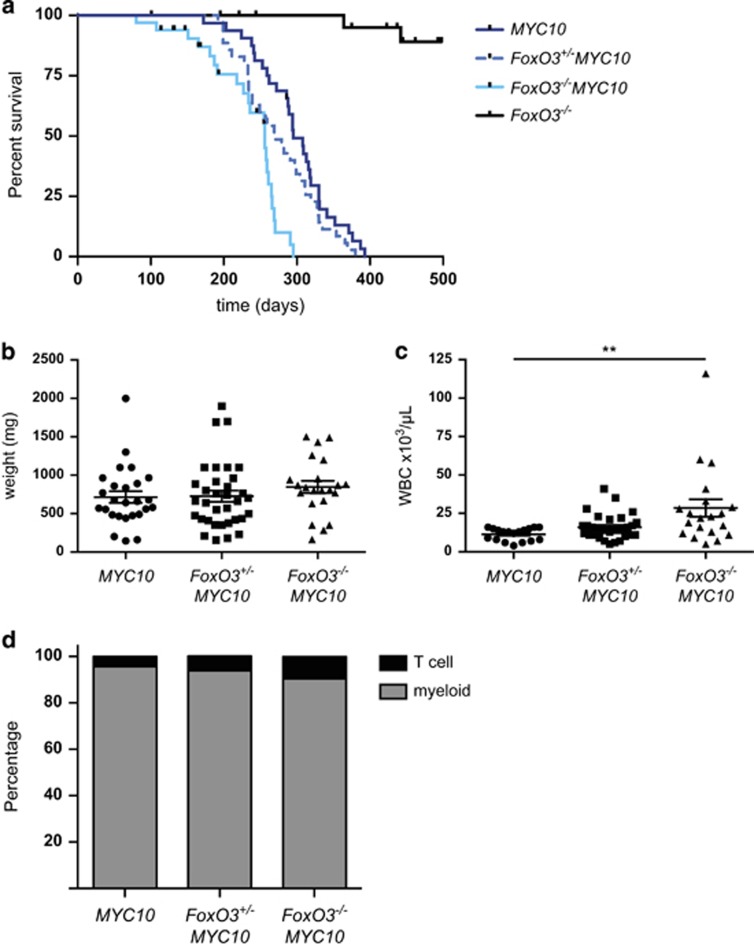
Loss of FoxO3 accelerates Myc-induced myeloid tumorigenesis. (**a**) Kaplan–Meier survival curves for *MYC10* (*n*=32, median survival 295 days), *FoxO3*^*+/−*^*MYC10* (*n*=35, median survival 270 days), *FoxO3*^−/−^*MYC10* (*n*=33, median survival 256 days) and *FoxO3*^−/−^ (*n*=24). Non-tumour-related deaths have been censored. Tumorigenesis is significantly accelerated in *FoxO3*^−/−^*MYC10 versus* *MYC10* mice, *P*<0.0001, log-rank test. (**b**) Spleen weights and (**c**) WBC counts of sick mice. Leukocytes were elevated in *FoxO3*^−/−^*MYC10* mice, **P<0.01, Student's t-test. (**d**) Proportions of myeloid and T-cell tumours in sick mice. Tumour cell suspensions were stained for cell surface markers and analysed by FACS. *n*=22–32

vavP-*MYC*10 (hereafter *MYC10*) transgenic mice develop macrophage tumours with a median onset of approximately 300 days. Lymphomagenesis was significantly accelerated by the loss of FoxO3, all *FoxO3*^−/−^*MYC10* mice having succumbed to disease by 300 days compared with 400 days for the *MYC10* mice (Figure 1a) (median survival of *FoxO3*^*−/−*^*MYC10* 256 days *versus* 295 days for *MYC10*; *P*<0.0001). Comparison of spleen weights and white blood cell (WBC) counts of tumour-bearing mice showed that the tumour burden was higher in the *FoxO3*^*−/−*^*MYC10* mice (Figures 1b and c). The tumour phenotype (Figure 1d) was comparable between the cohorts, being predominantly monocyte/macrophage tumours (Mac1^+^F4/80^+^) with a small proportion of T-cell tumours (Thy1^+^CD3^+^ and variable CD4 and CD8 expression), as previously reported for *MYC10* mice.^32^ Tumours of all genotypes were transplantable in C57BL/6 recipients (*n*=9–12 independent tumours).

**Figure 2:**
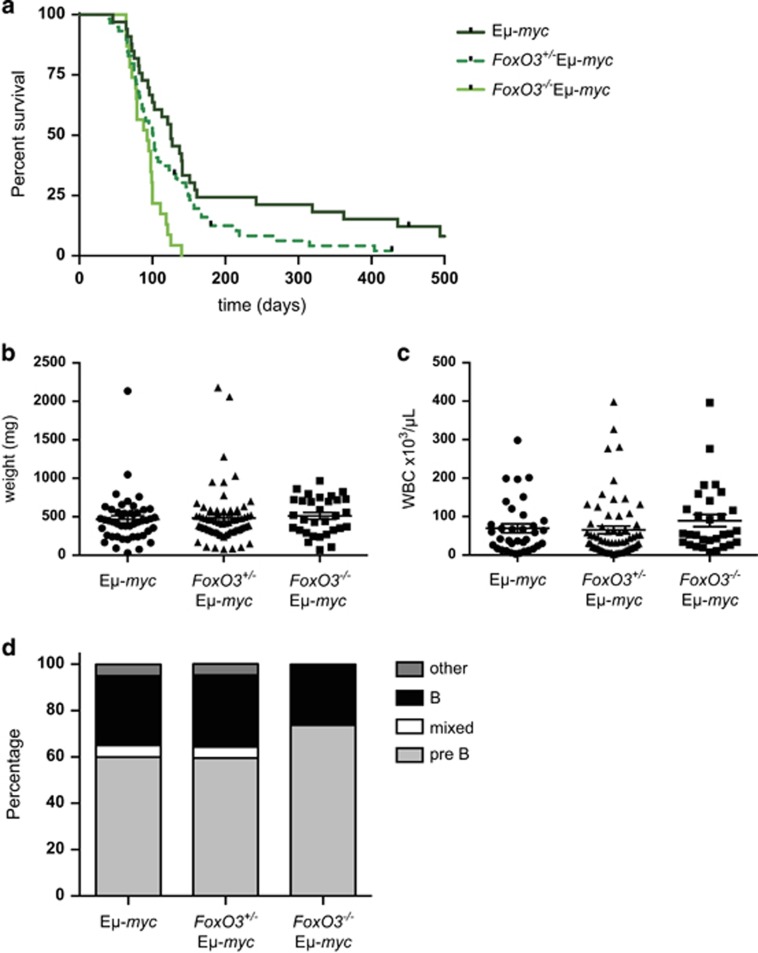
Loss of FoxO3 accelerates Myc-induced lymphomagenesis. (**a**) Kaplan–Meier survival curves for Eμ-*myc* (*n*=33, median survival 125 days), *FoxO3*^*+/−*^ Eμ-*myc* (*n*=59, median survival 101 days) and *FoxO3*^−/−^ Eμ-*myc* (*n*=23, median survival 93 days). Non-tumour-related deaths have been censored. Lymphomagenesis is significantly accelerated by loss of FoxO3. *FoxO3*^−/−^ Eμ-*myc versus* Eμ-*myc* mice, *P*<0.0001; *FoxO3*^*+/−*^ Eμ-*myc versus* Eμ-*myc* mice, *P*=0.0502; *FoxO3*^−/−^ Eμ-*myc versus* *FoxO3*^*+/−*^ Eμ-*myc*, *P*=0.0076; log-rank test. (**b**) Spleen weights and (**c**) WBC counts of sick mice. (**d**) Proportions of pre-B, mixed (pre-B/B), B and other lymphoid (T or primitive) tumours in sick mice; determined by staining for cell surface markers and FACS analysis. *n*=20–42

When we assessed the impact of FoxO3 loss on lymphomagenesis in Eμ-*myc* mice, again we found a significant acceleration in morbidity. The median survival of *FoxO3*^*−/−*^Eμ-*myc* mice was 93 *versus* 125 days for Eμ-*myc* mice (*P*<0.0001), with no *FoxO3*^*−/−*^Eμ-*myc* mice surviving beyond 150 days (Figure 2a). Tumour-bearing mice typically presented with enlarged spleen, lymph nodes and/or thymus, with no significant difference in lymphoma burden between genotypes (Figures 2b and c). Although Eμ-*myc* mice generally succumb to sIgM^−^B220^+^ pre-B lymphomas or sIgM^+^B220^+^ B-cell lymphomas,^33, 34^ certain crosses have led to changes in tumour phenotype, either predominantly B-cell lymphomas (e.g., in p53^+/−^/Eμ-*myc* and vavP-*Mcl-1*/Eμ-*myc* mice),^35, 36^ or a complete change in immunophenotype (e.g., in Eμ-*bcl-2*/ Eμ-*myc* and Eμ-v-*abl*/ Eμ-*myc* mice).^37, 38^ However, immunophenotyping of the *FoxO3*^−/−^Eμ-*myc* lymphomas revealed no major difference in the proportion of pre-B *versus* B-cell tumours compared with Eμ-*myc* lymphomas (Figure 2d).

### Haemopoietic homeostasis is perturbed by loss of FoxO3

**Figure 3:**
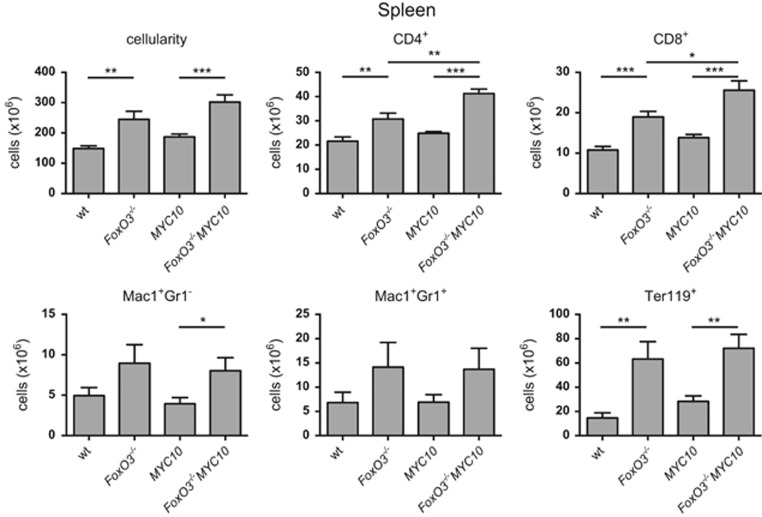
Loss of FoxO3 increases myeloid and T-cell populations in *MYC10* mice. Enumeration of total leukocytes and indicated populations in the spleen of preneoplastic 8-week-old male mice (*n*=8–9 per genotype). Bars represent mean±S.E.M.; statistical significance is shown only for WT *versus* *FoxO3*^−/−^, *MYC10 versus* *FoxO3*^−/−^*MYC10* and *FoxO3*^−/−^ *versus* *FoxO3*^−/−^*MYC10* (*P<0.05, **P<0.01, ***P<0.001, Student's *t*-test). See also Supplementary Table S1 and Supplementary Figure 1

To determine whether loss of FoxO3 alters the preneoplastic phenotype of *MYC10* mice, which could contribute to the enhanced tumorigenesis, we compared the composition of blood and haemopoietic tissues of healthy mice at 8 weeks of age (Figure 3, Supplementary Figure 1 and Supplementary Table 1).

Perhaps surprisingly, loss of FoxO3 alone had a greater impact on haemopoietic homeostasis than expression of the *MYC10* transgene, which provokes only a mild preneoplastic phenotype.^32^ The *FoxO3*^−/−^ mice had significantly increased T cells in the spleen and blood, but not in lymph nodes. Herold *et al.*^39^ have also reported an increase in splenic T cells using the same *FoxO3*^−/−^ mice, although increased T-cell numbers were not observed in an earlier study using a different line.^40^ Consistent with previous reports, the *FoxO3*^−/−^ mice also had increased myeloid cells in the spleen and bone marrow,^21, 41, 42^ reduced recirculating B cells in the bone marrow^43^ and elevated immature erythroid cells (Ter119^+^), presumably due to impaired maturation.^44, 45^

In general, the phenotype of *FoxO3*^−/−^*MYC10* mice resembled that of *FoxO3*^−/−^ mice. However, in the spleen, there was an additional increase in the number of T cells in *FoxO3*^−/−^*MYC10* mice compared with *FoxO3*^−/−^ mice (Figure 3). Significant increases in myeloid populations were also observed in the blood, spleen and bone marrow, in particular Mac1^+^Gr1^−^ cells, which were elevated approximately 2- to 2.5-fold in each organ (Figure 3, Supplementary Figure 1). As the majority of tumours arising in the *MYC10* mice are Mac1^+^Gr1^−^, it is likely that the increase in this population in *FoxO3*^−/−^*MYC10* mice is a factor in the acceleration of tumour onset.

**Figure 4:**
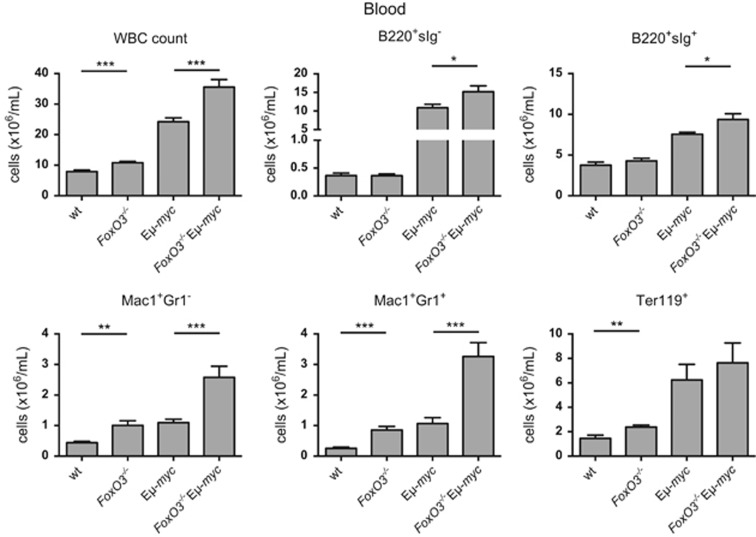
Loss of FoxO3 increases myeloid and B-cell populations in the blood of Eμ-*myc* mice. Total WBC count and quantification of the indicated populations in the blood of preneoplastic 4-week-old male mice (*n*=8–9 per genotype). Bars represent mean±S.E.M.; statistical significance is shown only for WT *versus* *FoxO3*^−/−^ and Eμ-*myc versus* *FoxO3*^−/−^ Eμ-*myc* (*P<0.05, **P<0.01, ***P<0.001, Student's *t*-test). See also Supplementary Table S2 and Supplementary Figure 2

Haemopoietic analysis of pre-leukaemic *FoxO3*^−/−^Eμ-*myc* mice and littermates was performed at 4 weeks of age (Figure 4, Supplementary Figure 2 and Supplementary Table 2). Loss of FoxO3 resulted in increased myeloid cells in the blood, spleen and bone marrow of Eμ-*myc* mice, as in *MYC10* mice, but no significant differences were seen in T-cell numbers. As Eμ-*myc* and *FoxO3*^−/−^Eμ-*myc* mice develop pre-B or B-cell lymphomas, the impact of FoxO3 loss on B lymphopoiesis was of particular interest. As documented previously,^46^ young preneoplastic Eμ-*myc* mice had a greatly expanded pre-B-cell (B220^+^IgM^−^IgD^−^) population, with 15- to 20-fold increases in the spleen, blood and lymph nodes, and there was a modest further increase in the blood of *FoxO3*^−/−^Eμ-*myc* mice.

### Cell cycle analysis

Deregulation of c-*myc* promotes tumorigenesis through altered expression of genes regulating cell cycling and proliferation.^47^ Loss of FoxO may be expected to amplify the cell cycle impact of *myc*, as FoxO3 regulates genes that promote cell cycle arrest.^6, 48^ We tested this possibility in preneoplastic populations, to avoid the complication of additional oncogenic mutations.

**Figure 5:**
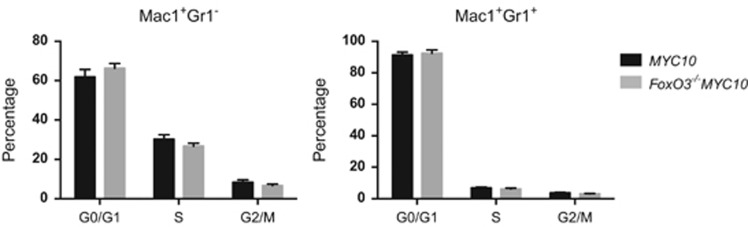
Loss of FoxO3 has no impact on the cycling of preneoplastic *MYC10* myeloid cells. Bone marrow from *MYC10* and *FoxO3*^−/−^*MYC10* mice was surface stained with Mac1 and Gr1 before fixation. Cell cycle analysis of macrophages (Mac1^+^Gr1^−^) and granulocytes (Mac1^+^Gr1^+^) was then performed following incubation with PI/RNase staining solution. Data represent mean±S.E.M.; *n*= 6–7 per genotype

As the *MYC10* and *FoxO3*^−/−^*MYC10* mice are predisposed to myeloid tumours, we analysed the cell cycle profiles of bone marrow macrophages and granulocytes from healthy young mice. No differences in the cell cycle distribution were detectable in either Mac1^+^Gr1^−^ or Mac1^+^Gr1^+^ cells (Figure 5).

**Figure 6:**
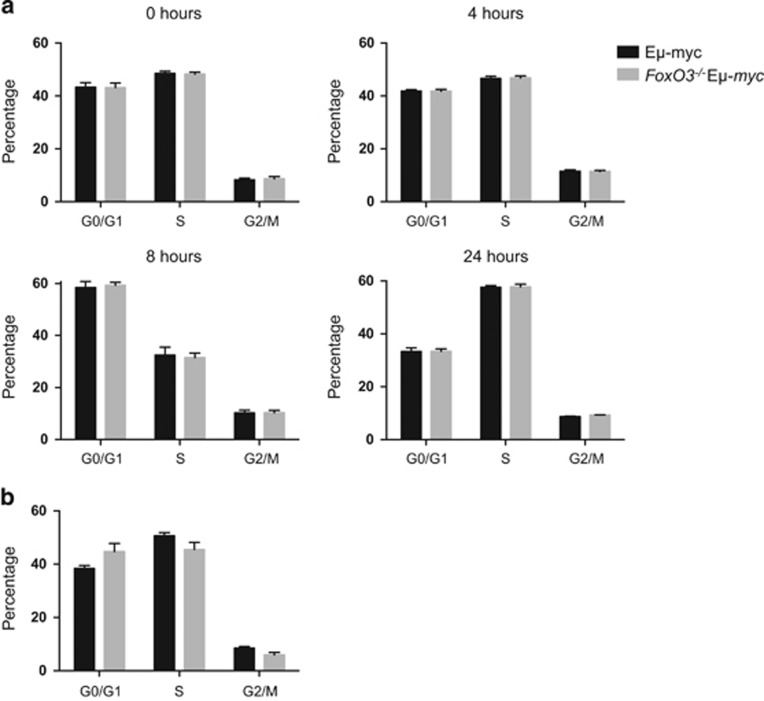
Loss of FoxO3 has no impact on the cycling of preneoplastic and neoplastic Eμ-*myc* pre-B cells. (**a**) Pre-B cells (B220^+^sIg^−^) were purified from the bone marrow of 4-week-old Eμ-*myc* and *FoxO3*^−/−^ Eμ-*myc* mice by flow cytometry and cultured without cytokines. Cell cycle analysis was performed using Nicoletti staining at the indicated time points and flow cytometry. Cell cycle phases were determined using the Watson pragmatic model within the flow cytometry data analysis software FlowJo. Bars represent mean±S.E.M.; *n*= 4–5 per genotype. (**b**) Pre-B/B cells were purified from tumours of Eμ-*myc* and *FoxO3*^−/−^ Eμ-*myc* mice using CD19 beads and MACS columns. Cells were fixed then stained with PI/RNase staining solution. Cell cycle populations were analysed by FACS. Bars represent mean±S.E.M.; *n*= 5 per genotype

For the Eμ-*myc* and *FoxO3*^−/−^ Eμ-*myc* mice, preneoplastic pre-B cells were analysed immediately after isolation from the bone marrow and also during culture in simple medium lacking exogenous cytokines. However, there was no difference between Eμ-*myc* and *FoxO3*^−/−^Eμ-*myc* pre-B-cell cycle profiles either before culture (first panel, Figure 6a) or at any of the time points analysed (4 h, 8 h, 24 h; Figure 6a). Furthermore, tumour cells isolated from Eμ-*myc* and *FoxO3*^−/−^Eμ-*myc* mice had similar cell cycle profiles (Figure 6b).

### Impact of loss of FoxO3 on apoptosis of Myc-overexpressing cells

The proliferative effect of Myc overexpression is partially counteracted by Myc-driven apoptosis.^49, 50^ We therefore investigated whether loss of FoxO3 inhibited apoptosis of cells overexpressing Myc. We reasoned that the impact of loss of FoxO3 may become more apparent during culture, as the deficiency in cytokine signalling would normally inactivate AKT and activate FoxO3.

As expression of the vavP-*MYC* transgene is highest in thymocytes,^32^ we isolated the four major thymocyte populations by flow cytometry from wild-type (WT), *FoxO3*^−/−^, *MYC10* and *FoxO3*^−/−^ *MYC10* mice and cultured them in simple medium (i.e., in the absence of cytokines) either immediately (Supplementary Figure 3A) or following exposure to 1.25 Gy *γ*-irradiation (Supplementary Figure 3B). For double-positive thymocytes, the most sensitive population, there was a trend toward an increased viability of *FoxO3*^−/−^ compared with WT cells under these conditions, but this was not apparent on the *MYC10* background. Loss of FoxO3 also enhanced the survival of peripheral T-cell blasts following removal of interleukin 2 (IL-2), as noted previously,^39^ and this was evident even in the face of expression of the *MYC10* transgene (Supplementary Figure 3C).

**Figure 7:**
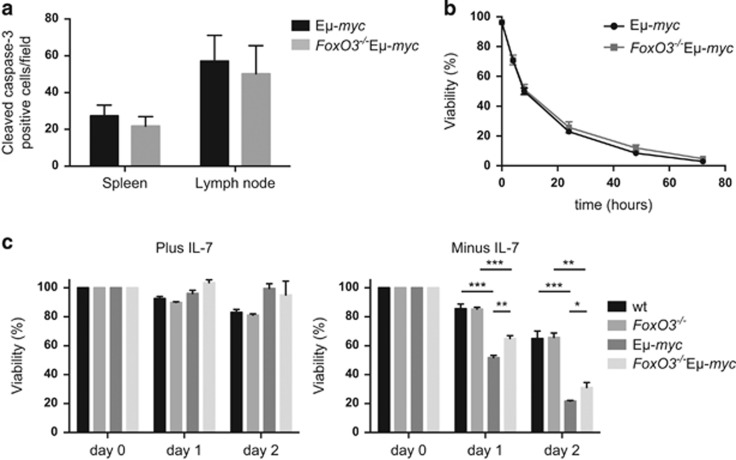
Impact of loss of FoxO3 on the apoptosis of preneoplastic and neoplastic Eμ-*myc* cells. (**a**) Quantification of cleaved caspase-3 positive cells in the spleen and lymph node of sick Eμ-*myc* and *FoxO3*^−/−^ Eμ-*myc* mice. For each tumour section (blinded as to the genotype), cleaved caspase-3-positive cells were enumerated from images of three independent fields of view at x200 magnification. Bars represent mean±S.E.M.; *n*=4–6 per genotype. (**b**) Spontaneous death of FACS purified preneoplastic pre-B cells (B220^+^sIg^-^) from 4-week-old Eμ-*myc* and *FoxO3*^−/−^ Eμ-*myc* mice cultured in simple medium (no cytokines) was assessed by staining with PI and annexin V followed by FACS analysis (*n*=5–6 per genotype). Values represent mean±S.E.M. (**c**) Enhanced survival upon IL-7 withdrawal of cultured *FoxO3*^−/−^ Eμ-*myc* pre-B cells. Pre-B cells isolated from bone marrow of mice of the indicated genotypes were cultured in the presence of 2% IL-7 supernatant for 5 days. Survival following removal of IL-7 was determined at the indicated time points by flow cytometry, with data normalised to the viability of each culture when IL-7 was withdrawn, day 0. Bars represent mean±S.E.M.; *n*=3–4 mice for each genotype. *FoxO3*^−/−^ Eμ-*myc* pre-B cells were significantly more viable than Eμ-*myc* pre-B cells on day 1 (*P*=0.0028) and day 2 (*P*=0.0250). *P<0.05, **P<0.01, ***P<0.001, Student's *t*-test

Apoptosis was also assessed for B lymphoid cells from Eμ-*myc* and *FoxO3*^*−/−*^Eμ-*myc* mice. Frank tumours had comparable numbers of apoptotic cells (Figure 7a and Supplementary Figure 4). When pre-leukaemic pre-B cells sorted from bone marrow were cultured in the absence of cytokines, no significant survival advantage could be detected for those lacking FoxO3 (Figure 7b). However, when IL-7 was removed from IL-7-supported cultures of pre-B cells, loss of FoxO3 significantly enhanced the survival of Eμ-*myc* (but not WT) pre-B cells (Figure 7c). As IL-7 has a critical role in early B lymphopoiesis, this protective effect may account for the modest increase in pre-B cells in *FoxO3*^*−/−*^Eμ-*myc* compared with E*μ*-*myc* mice (Figure 4, Supplementary Table 2).

### Loss of FoxO3 does not select against inactivation of the p53 pathway

**Figure 8:**
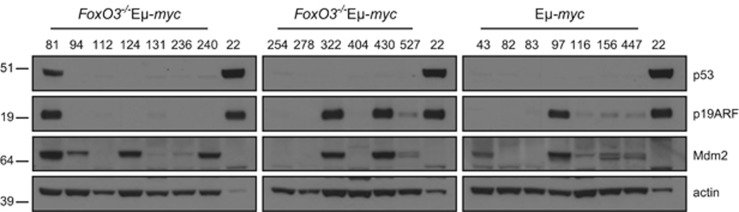
Loss of FoxO3 does not relieve the pressure for mutation of the p53 pathway in Eμ-*myc* lymphomagenesis. Western blot analysis of p53, p19ARF and Mdm2 in the indicated Eμ-*myc* and *FoxO3*^−/−^ Eμ-*myc* lymphomas; Eμ-*myc* #22 (right-hand lane on each gel), a known p53 mutant cell line, was included as a positive control. MW markers are indicated (kDa). High expression of p19ARF is indicative of p53 mutation (e.g., *FoxO3*^−/−^ Eμ-*myc* #81) or loss (e.g., *FoxO3*^−/−^ Eμ-*myc* #322, #430 and Eμ-*myc* #97). High expression of Mdm2 promotes ubiquitinylation and degradation of p53, thereby abrogating p53-mediated apoptosis

Lymphomas arising in Eμ-*myc* mice commonly bear mutations that inactivate the p19Arf/Mdm2/p53 pathway, either by *p53* mutation or deletion, *p19Arf* deletion, or overexpression of Mdm2.^51^ Although expression of dominant-negative FoxO (dnFoxO) reduced selective pressure to inactivate the p19Arf/p53 pathway during lymphomagenesis in Eμ-*myc* mice,^26^ this was not the case for *FoxO3*^−/−^Eμ-*myc* lymphomas (Figure 8). One *FoxO3*^−/−^Eμ-*myc* tumour (# 81) had high levels of p53 and p19Arf, suggestive of a p53 inactivating mutation (as carried by the positive control, Eμ-*myc* lymphoma #22). Certain other *FoxO3*^−/−^Eμ-*myc* lymphomas (#322 and #430) had high levels of p19Arf but expressed no p53, apparently because of upregulation of Mdm2. Two, perhaps three, other *FoxO3*^−/−^Eμ-*myc* lymphomas lacking p53 expression had elevated Mdm2 but did not express p19Arf (#124, #240 and perhaps #94), suggestive of p19Arf deletion, as observed previously.^51^ Thus, overall, 5 or 6 of 13 (38–46%) *FoxO3*^−/−^E*μ*-*myc* lymphomas analysed showed evidence of loss of p53 or Arf function, comparable to the 13/25 (52%) reported by Eischen *et al.*^51^ Thus, the absence of FoxO3 alone appears insufficient to select against inactivation of the p53 pathway.

## Discussion

We have demonstrated, for the first time, that loss of a single FoxO transcription factor, can promote lymphomagenesis. Whereas Paik *et al.*^25^ found that complete loss of FoxO activity (both alleles of FoxO1, FoxO3 and FoxO4) is required for lymphomagenesis, we have shown using two different mouse models that loss of FoxO3 alone suffices to accelerate Myc-driven lymphomagenesis. In the absence of FoxO3, myeloid tumours arose more rapidly in vavP-*MYC*10 mice and pre-B and B lymphomas arose more rapidly in Eμ-*myc* transgenic mice (Figures 1 and 2). Unsurprisingly, the reduction in tumour latency was not as great as that observed when a dominant-negative FoxO construct was used to inhibit all FoxOs in the Eμ-*myc* model.^25^ Deficiency in a single FoxO does not have an observable effect in all tumour models, however, as loss of FoxO3 or FoxO4 does not accelerate or increase the incidence of DMBA-induced tumorigenesis.^25^

The larger population of myeloid cells in FoxO3-deficient *MYC10* mice is likely to be a significant factor contributing to accelerated myeloid tumour formation in this model (Figure 3,Supplementary Figure 1 and Supplementary Table 1). In the Eμ-*myc* model, expansion of the pre-B-cell pool, although modest, may contribute to accelerated lymphoma development in the absence of FoxO3.

Several factors may contribute to increasing the size of the susceptible populations. The absence of FoxO3 had no discernible impact on the cycling of pre-leukaemic myeloid cells isolated from *MYC10* mice (Figure 5) or of pre-leukaemic or neoplastic B lymphoid cells taken from Eμ-*myc* mice (Figure 6). Presumably other FoxOs can compensate in this role – FoxO1, in particular, was readily detected in lymphoid cells from Eμ-*myc* and vavP-*MYC* mice (Supplementary Figure 5), as reported previously for WT lymphoid cells.^16^ Previous studies have reported that FoxO3-deficiency increased the viability of mast cells and thymocytes cultured in the absence of cytokines^39, 52^ and we were able to demonstrate enhanced viability of certain T-cell populations from vavP-*MYC10* mice, particularly after cytokine removal (Supplementary Figure 3). Similarly, lack of FoxO3 enhanced the viability of cultured Eμ-*myc* pre-B cells following IL-7 removal (Figure 7c). Reduced apoptosis has also been documented in dnFoxO-expressing Eμ-*myc* lymphoma cells.^26^

Activation of p53 is a principal route for Myc-induced apoptosis. Myc upregulates p19Arf expression, probably indirectly, and p19Arf binds to and neutralises Mdm2, thereby preventing inhibition and degradation of p53 and facilitating expression of pro-apoptotic p53 targets, BH3-only proteins Puma and Noxa and, in a negative feedback loop, Mdm2.^53^ Although expression of dnFoxO reduced the selective pressure to inactivate the p19Arf/p53 pathway during lymphomagenesis in Eμ-*myc* mice,^26^ this was not the case for *FoxO3*^−/−^Eμ-*myc* lymphomas (Figure 8).

In summary, we have demonstrated using two different mouse models that FoxO3 has a significant tumour-suppressor function in the context of Myc-driven lymphomagenesis. Although our data suggest a role for FoxO3-mediated inhibition of apoptosis in restraining Myc-driven tumour development, we suspect that loss of FoxO3 may also be affecting other tumour-suppressive functions for which FoxO1/4 cannot fully compensate. Of note, the tumour-suppressive activity of p53 does not involve its capacity to promote apoptosis or cell cycle arrest.^54, 55^

## Materials and Methods

### Mice

Experimental protocols involving the use of mice were approved by the Walter and Eliza Hall Institute's Animal Ethics Committee. All mice were on a C57BL/6 background and were bred at the Walter and Eliza Hall Institute (WEHI). To generate *FoxO3*^−/−^*MYC10* mice, *FoxO3*^*+/−*^ (ref. 21) females were crossed with *MYC10*^hom^^32^ males (i.e., homozygous for the *MYC10* transgene) then offspring were interbred. *FoxO3*^−/−^ Eμ-*myc* mice were produced by mating *FoxO3*^+/−^ females with Eμ-*myc*^33^ males followed by interbreeding of offspring. Analysis was performed on healthy young mice (4 or 8 weeks of age) and cohorts of mice were aged to ethical end point.

### Haemopoietic analysis

Blood counts and composition were determined using an ADVIA 2120 haematology analyser (Siemens, Erlangen, Germany). In addition, for preneoplastic analysis, the remaining blood was depleted of red blood cells using 0.168 M ammonium chloride and cell composition determined by flow cytometry, as below. Single-cell suspensions were prepared from spleen, thymus, lymph nodes (axillary, brachial, inguinal) and bone marrow and leukocytes enumerated with a CASY Cell Counter (Scharfe System GmbH, Reutlingen, Germany). Cell composition was determined by staining with fluorochrome-labelled surface marker-specific monoclonal antibodies followed by fluorescence-activated cell sorting (FACS) analysis using an LSRI (BD Biosciences, Franklin Lakes, NJ, USA). Data were processed using FlowJo Version 9.3.2 (TreeStar, Ashland, OR, USA). The monoclonal antibodies, produced and labelled with fluorescein isothiocyanate (FITC), R-phycoerythrin or allophycocyanin at WEHI, were: RB6-8C5, anti-Gr1; MI/70, anti-Mac1; H129.19, anti-CD4; YTS169, anti-CD8; Ter119, anti-erythroid marker; RA3-6B2, anti-CD45R-B220; 5.1, anti-IgM; 11-26C, anti-IgD; T24-31, anti-Thy1.

### Cell cycle analysis

Pre-B cells (B220^+^IgM^−^IgD^−^) were purified from the bone marrow of 4-week-old mice by flow cytometry, or by using CD19 MicroBeads (Miltenyi Biotech, Bergisch Gladbach, Germany) and MS columns (Miltenyi Biotech) according to the manufacturer's protocols. The Nicoletti assay^56^ was used for cell cycle analysis of the purified pre-B cells – cells were resuspended in 0.1% sodium citrate, 0.1% Triton X and 50 *μ*g/ml propidium iodide (PI) and incubated for 30 min on ice before FACS analysis. Alternatively, mixed cell populations, such as bone marrow, were stained with surface markers for the population of interest before fixation and permeabilisation with the Transcription Factor Staining Buffer Set (eBioscience, San Diego, CA, USA). Cells were then stained with PI/RNase staining solution (Cell Signaling Technology, Danvers, MA, USA) for 30 min before analysis on an LSR II flow cytometer (BD Biosciences). Cell cycle profiles were characterised using FlowJo using the Watson pragmatic model (see above).

### Survival assays

Thymocyte populations or pre-B cells isolated by flow cytometry were cultured at 0.2–0.5x10^6^ cells/ml in high-glucose Dulbecco's Modified Eagle's medium supplemented with 10% foetal calf serum (JRH Biosciences, Brooklyn, VIC, Australia), 50 *μ*M 2-mercaptoethanol (2-ME; Sigma-Aldrich, St Louis, MO, USA) and 100 *μ*M asparagine (Sigma-Aldrich) without additional cytokines. In addition, thymocytes were treated before culture with 1.25 Gy *γ*-irradiation. Cell viability was determined by staining with FITC-conjugated annexin V and PI followed by flow cytometry. Specific viability was calculated at each time point as (viability of treated cells/viability of untreated cells) x 100%.

### Cytokine withdrawal

Pre-B cells (B220^+^IgM^−^IgD^−^) purified from bone marrow by flow cytometry were cultured at a starting density of 1x10^6^/ml in Iscove's modified Dulbecco's medium supplemented with 1x GlutaMAX, 1 mM sodium pyruvate, 0.1 mM nonessential amino acids, 10 mM HEPES pH 7.4 (all media supplements from Life Technologies, Carlsbad, CA, USA), 50 *μ*M 2-ME, 10% foetal calf serum and 2% mIL-7 supernatant (produced by transfected x63/0 hybridoma cells).^57^ Cultures were split if required. After 5 days, cells were washed three times to remove IL-7, then replated at 0.3x10^6^/ml with or without IL-7 for 2 days. Viability was determined by flow cytometry as above.

T-cell blasts were generated by stimulation of splenocytes (2x10^6^/ml) for 3 days with 1% mIL-2 supernatant^57^ and 2 *μ*g/ml concanavalin A (Sigma-Aldrich) in cell culture medium as for survival assays above. Cells were then washed three times to remove concanavalin A and cultured with IL-2 alone for 24 h. To set up the death assay, cells were washed three times to remove IL-2 then cultured with or without IL-2 for 2 days. Viability was determined by flow cytometry as above.

### Cleaved caspase-3 immunohistochemistry

Spleens and lymph nodes from lymphoma-bearing mice were fixed in 10% formalin then embedded in paraffin. Sections were stained for cleaved caspase-3 using the SignalStain Apoptosis (Cleaved Caspase-3) IHC Detection Kit (Cell Signaling Technology) according to the manufacturer's protocol. Stained slides were imaged (blinded as to genotype) using an Olympus BX43 microscope (Olympus, Tokyo, Japan) and Olympus DP72 camera at x200 magnification.

### Immunoblotting

Protein extracts were prepared by lysis in RIPA buffer (300 mM NaCl, 2% IGEPAL CA-630, 1% deoxycholic acid, 0.2% SDS, 100 mM Tris-HCl pH 8.0) containing complete ULTRA protease inhibitors (Roche, Basel, Switzerland). Western blots were carried out using 30 *μ*g total protein per sample run on NuPAGE Bis-Tris gels (Life Technologies) and transferred to nitrocellulose membranes with an iBlot (Life Technologies) according to the manufacturer's protocols. Blots were probed with the following antibodies: p53 (FL-393, Santa Cruz Biotechnology, Santa Cruz, CA, USA); p19ARF (p19ARF exon 2, Rockland, Gilbertsville, PA, USA); Mdm2 (C-18, Santa Cruz Biotechnology); FoxO3 (75D8, Cell Signaling Technology); FoxO1 (C29H4, Cell Signaling Technology); and β-actin (clone AC-74, Sigma-Aldrich).

### Statistical analysis

GraphPad Prism (Version 6.0d, GraphPad Software, San Diego, CA, USA) was used to graph and statistically analyse data. Student's *t*-test (an unpaired two-tailed t-test) or the log-rank (Mantel–Cox) test, for Kaplan–Meier mouse survival curves, were used to determine statistical significance.

## Abbreviations

dnFoxO: dominant-negative FoxO
FACS: fluorescence-activated cell sorting
FoxO: Forkhead-box O class
HSC: haemopoietic stem cell
IHC: immunohistochemistry
IL-2: interleukin 2
IL-7: interleukin 7
PI: propidium iodide
sIg: surface immunoglobulin
WBC: white blood cell
WT: wild type.
