# Supplementary material for: FoxO3 suppresses Myc-driven lymphomagenesis
Source: Cell Death Dis. 2016 Jan 14;7(1):e2046–. doi: 10.1038/cddis.2015.396 (PMC4816178; doi:10.1038/cddis.2015.396)

Supplementary Figure 1

A

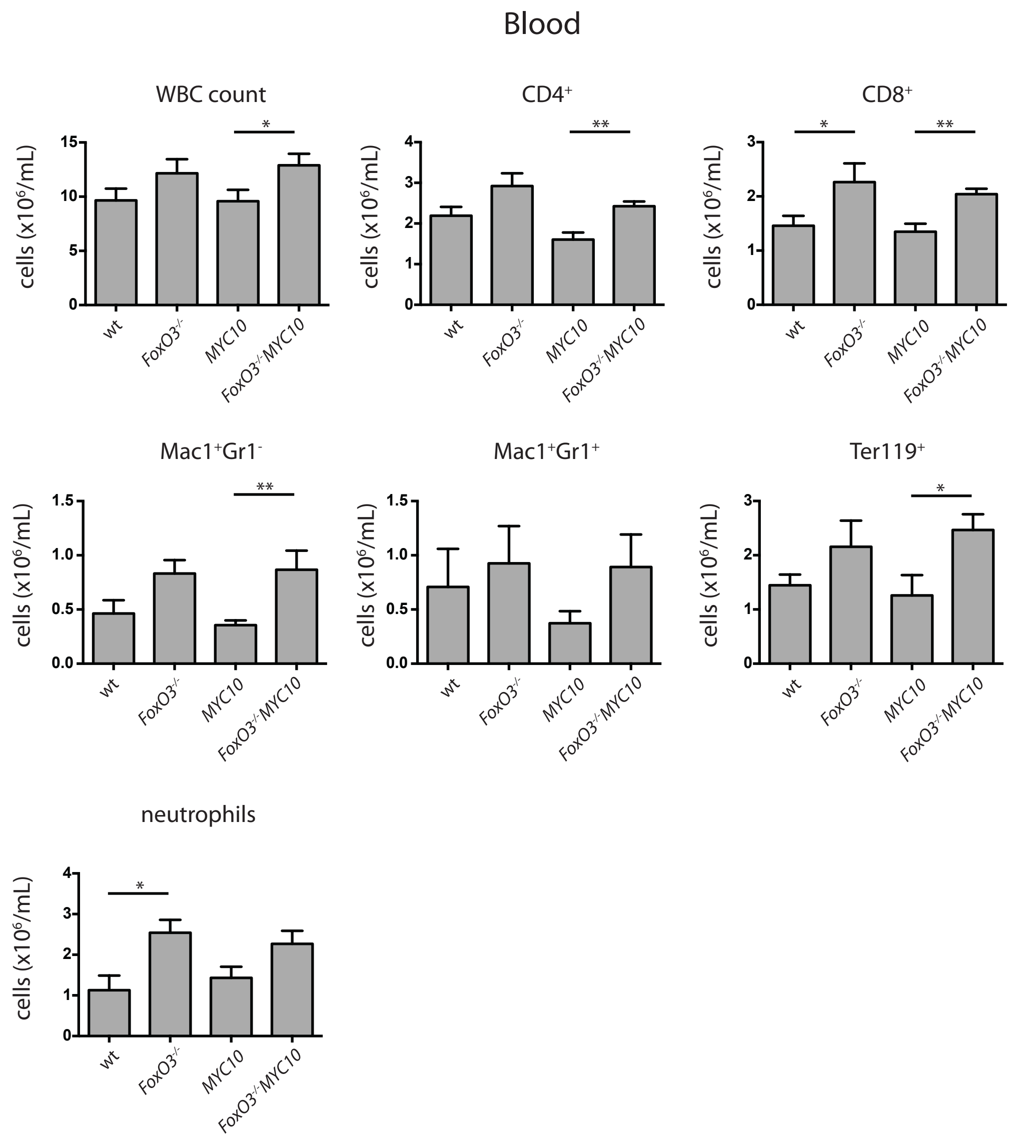

B

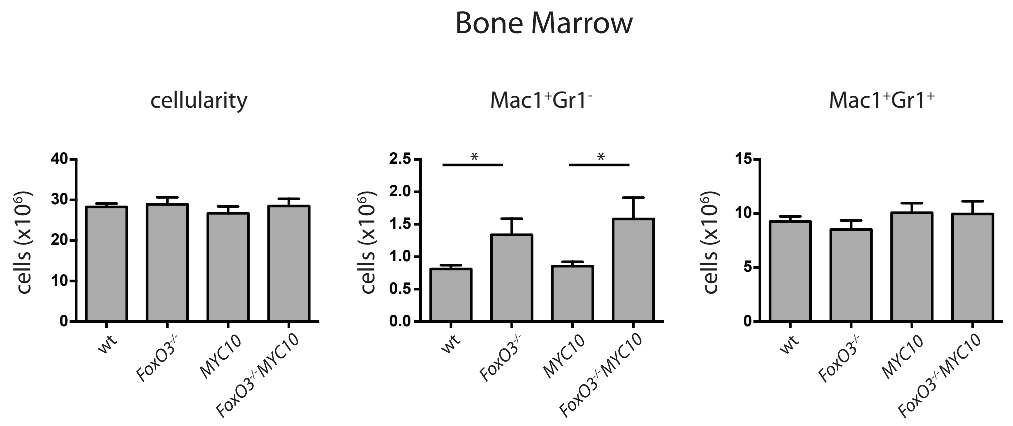

Supplementary Figure 2

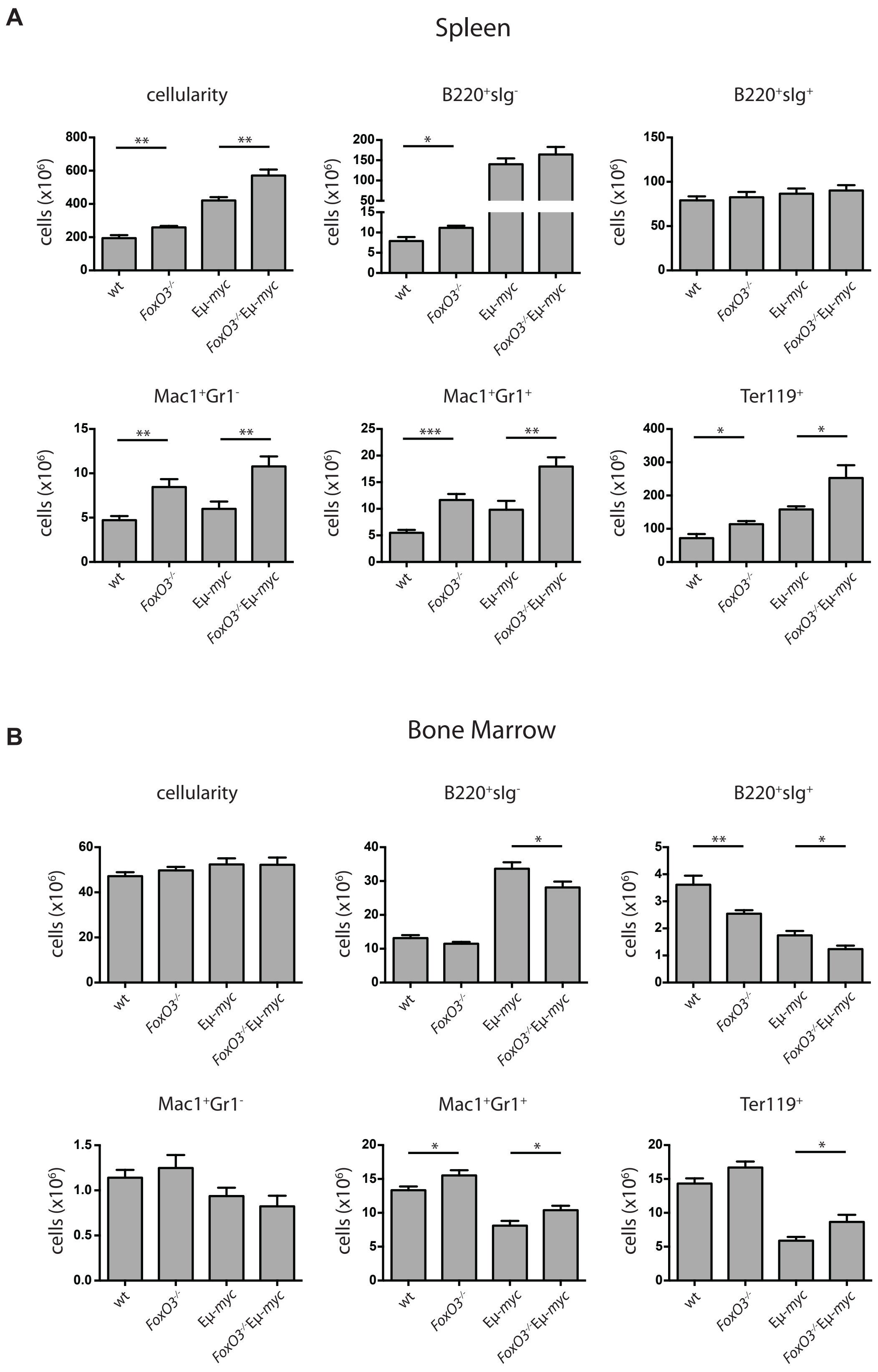

Supplementary Figure 3

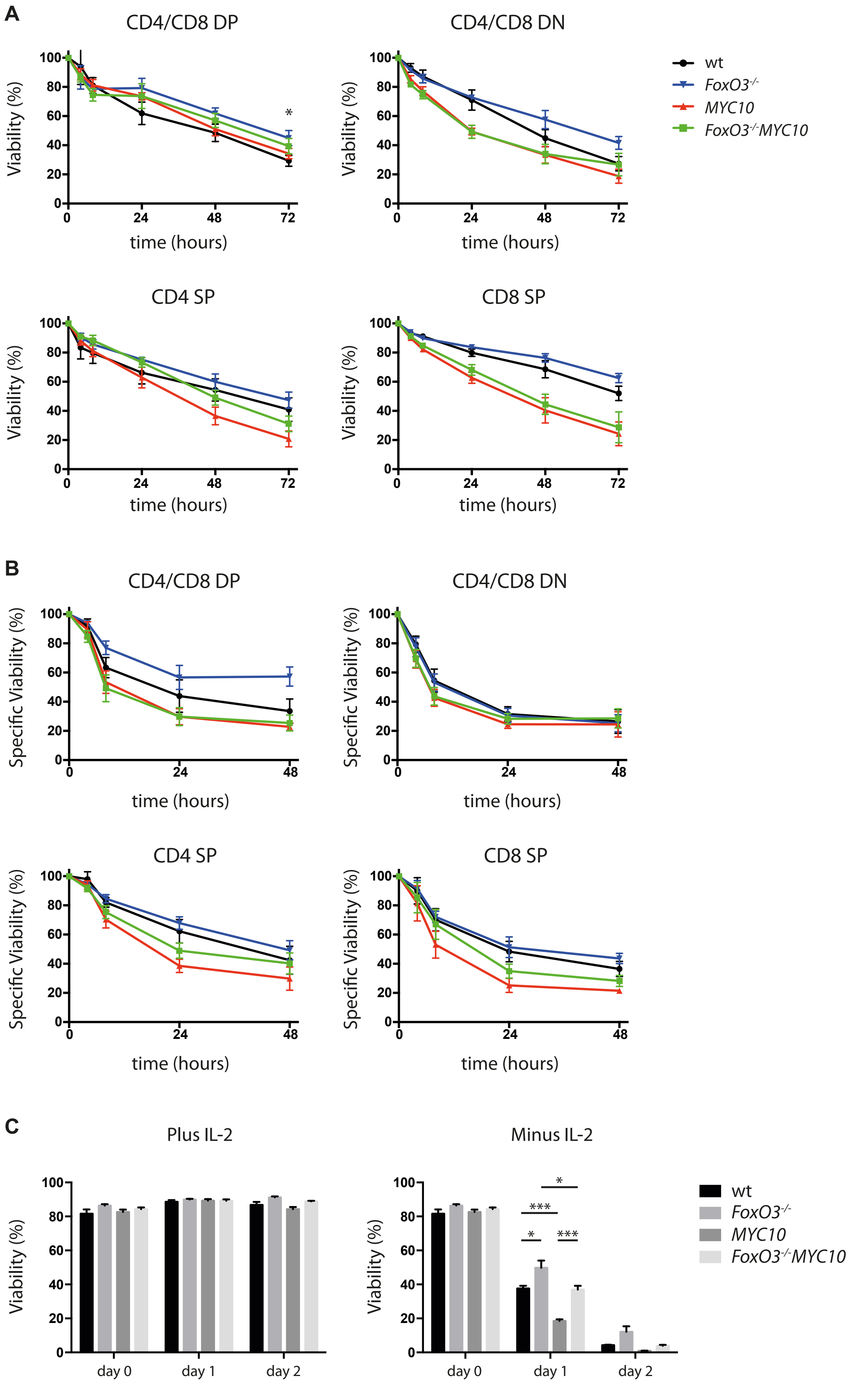

Supplementary Figure 4

Eμ-*myc* #301

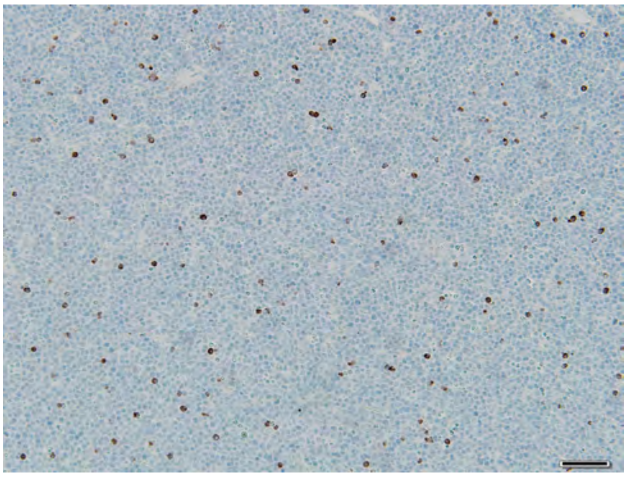

FoxO3$^{-/-}$Eμ-*myc* #404

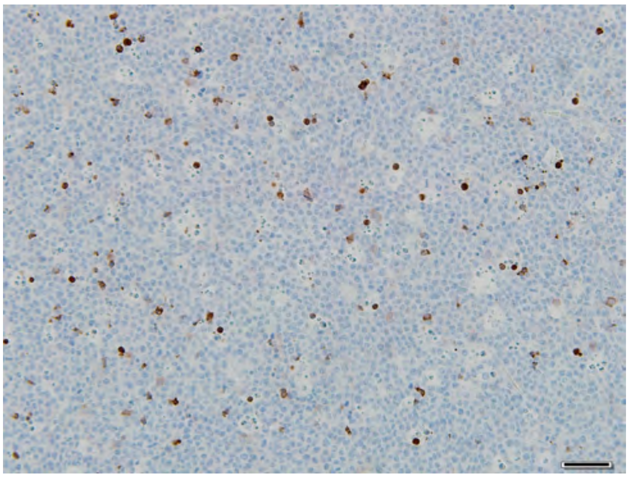

Supplementary Figure 5

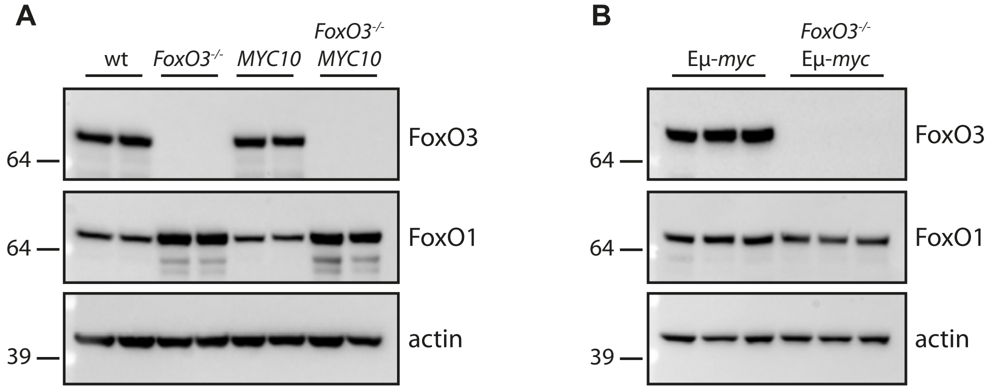

Supplement: Supplementary Figures [file cddis2015396x1.pdf]
