# Supplementary material for: FoxO3 suppresses Myc-driven lymphomagenesis
Source: Cell Death Dis. 2016 Jan 14;7(1):e2046–. doi: 10.1038/cddis.2015.396 (PMC4816178; doi:10.1038/cddis.2015.396)
Supplement: Supplementary Information [file cddis2015396x2.docx]

**Supplementary Information**

**Supplementary Figure 1** Loss of FoxO3 increases myeloid and T cell populations in *MYC10* mice. Enumeration of the indicated populations in the blood (A) and bone marrow (one femur) (B) of preneoplastic 8-week-old male mice (n=8-9 per genotype). Bars represent mean ± SEM; Statistical significance is shown only for WT vs. *FoxO3*^-/-^, *MYC10* vs. *FoxO3*^-/-^*MYC10* and *FoxO3*^-/-^ vs. *FoxO3^-/-^MYC10* (**P*<0.05, ***P*<0.01, Student’s t-test). See also Supplementary Table S1.

**Supplementary Figure 2** Impact of loss of FoxO3 on myeloid and B lymphoid populations in Eµ-*myc* mice. Enumeration of the indicated populations in the spleen (A) and bone marrow (both femurs) (B) of preneoplastic 4-week-old male mice (n=8-9 per genotype). Bars represent mean ± SEM; Statistical significance is shown only for WT vs. *FoxO3*^-/-^ and Eµ-*myc* vs. *FoxO3*^-/-^ Eµ-*myc* (**P*<0.05, ***P*<0.01, ****P*<0.001, Student’s t-test). See also Supplementary Table S2.

**Supplementary Figure 3** Impact of loss of FoxO3 on apoptosis of *MYC10* T cells. (A and B) Thymocyte populations were isolated by flow cytometry and cultured in media without additional cytokines (A) or treated with 1.25 Gy γ-irradiation (B) prior to culture. At the indicated time-points cells were stained with PI and annexin V then analysed by FACS (n = 4 per genotype). Values represent mean ± SEM. Statistical significance is shown only for WT vs. *FoxO3*^-/-^ and *MYC10* vs. *FoxO3^-/-^MYC10* (**P*<0.05, Student’s t-test). (C) Increased survival of T cell blasts following IL-2 withdrawal in T cell blasts lacking FoxO3. T cell blasts were generated from spleens of WT, *FoxO3*^-/-^, *MYC10* and *FoxO3^-/-^MYC10* mice by stimulation for 3 days with IL-2 and concanavalin A. Survival following removal of IL-2 and concanavalin A was determined at the indicated time points by flow cytometry. Bars represent mean ± SEM; n = 4 mice for each genotype. Statistical significance is shown only for day 1 (**P*<0.05, ***P*<0.01, ****P*<0.001, Student’s t-test), where T cell blasts were significantly more viable for *FoxO3*^-/-^ vs. WT (*P*=0.0368) and *FoxO3^-/-^MYC10* vs. *MYC10* (*P*=0.0003).

**Supplementary Figure 4** Apoptosis in Eµ-*myc* lymphomas is unaltered by lack of FoxO3. Cleaved caspase-3 staining was performed on spleen and lymph node sections from Eµ-*myc* and *FoxO3*^-/-^ Eµ-*myc* lymphoma bearing mice. Cleaved caspase-3 stained lymph node sections at 200x magnification. Scale bar = 100 µm.

**Supplementary Figure 5** Expression of FoxO1 and FoxO3. Western blot analysis of FoxO3 and FoxO1 expression in (A) total thymocytes from WT, *FoxO3*^-/-^, *MYC10* and *FoxO3^-/-^MYC10* mice (n = 2 for each genotype) and (B) FACS sorted bone marrow pre-B cells from Eµ-*myc* and *FoxO3*^-/-^ Eµ-*myc* mice (n = 3 for each genotype). MW markers are indicated (kDa). Note that FoxO1 expression is elevated in thymocytes lacking FoxO3 but not in pre-B cells lacking FoxO3.

**Table S1. Haemopoietic composition of *FoxO3*^-/-^*MYC10* mice**

**Organ/cell type WT *FoxO3*^-/-^ *MYC10* *FoxO3^-/-^MYC10***

**Peripheral blood** 9.7 ± 3.3 12.2 ± 3.4 9.6 ± 3.2 12.9 ± 3.0^

CD4^+^CD8^-^ 2.2 ± 0.6 2.9 ± 0.8 1.6 ± 0.5* 2.4 ± 0.3^^

CD4^-^CD8^+^ 1.5 ± 0.5 2.3 ± 0.9* 1.3 ± 0.4 2.0 ± 0.3*^^

B220^+^IgM^-^IgD^-^ 0.21 ± 0.15 0.17 ± 0.13 0.24 ± 0.15 0.30 ± 0.15

B220^+^ sIg^+^ 4.4 ± 1.9 4.7 ± 1.5 4.7 ± 1.6 5.8 ± 2.3

Mac1^+^Gr1^-^ 0.46 ± 0.37 0.83 ± 0.33 0.36 ± 0.13 0.87 ± 0.50^^

Mac1^+^Gr1^+^ 0.71 ± 1.05 0.93 ± 0.91 0.37 ± 0.34 0.89 ± 0.85

Ter119^+^ 1.5 ± 0.6 2.2 ± 1.3 1.3 ± 1.1 2.5 ± 0.8**^

**Spleen** 149 ± 28 245 ± 77** 187 ± 29* 302 ± 63***^^^

CD4^+^CD8^-^ 21.6 ± 5.3 30.7 ± 6.9** 24.8 ± 2.0 41.2 ± 4.9***^^^🞟🞟

CD4^-^CD8^+^ 10.8 ± 2.5 19.0 ± 3.9*** 13.8 ± 2.3* 25.6 ± 6.1***^^^🞟

B220^+^IgM^-^IgD^-^ 4.0 ± 2.3 6.9 ± 3.7 7.1 ± 3.6* 11.9 ± 5.5**^

B220^+^ sIg^+^ 88.1 ± 10.3 109 ± 26* 107 ± 12** 139 ± 29***^^🞟

Mac1^+^Gr1^-^ 5.0 ± 3.0 9.0 ± 6.5 4.0 ± 2.3 8.0 ± 4.5^

Mac1^+^Gr1^+^ 6.8 ± 6.4 14.1 ± 14.4 6.9 ± 4.6 13.7 ± 12.2

Ter-119^+^ 14.6 ± 12.6 63.2 ± 40.8** 28.3 ± 13.8* 72.1 ± 32.5***^^

**LN** 22.2 ± 9.2 20.6 ± 10.4 30.7 ± 9.2 28.3 ± 11.4

CD4^+^CD8^-^ 8.1 ± 2.7 7.7 ± 4.0 10.1 ± 2.6 9.4 ± 4.0

CD4^-^CD8^+^ 4.7 ± 1.8 5.4 ± 2.6 5.0 ± 1.3 6.2 ± 2.5

B220^+^IgM^-^IgD^-^ 0.38 ± 0.36 0.26 ± 0.25 0.76 ± 0.49 0.92 ± 0.58*🞟

B220^+^ sIg^+^ 8.4 ± 4.3 6.8 ± 3.4 12.7 ± 4.5 10.5 ± 3.9

**BM** 28.3 ± 2.5 28.9 ± 4.9 26.7 ± 5.2 28.5 ± 5.0

Ter-119^+^ 11.3 ± 4.6 14.2 ± 5.4 8.7 ± 2.9 11.3 ± 2.8

Thy1^+^ 0.68 ± 0.14 0.47 ± 0.18* 0.59 ± 0.30 0.50 ± 0.16*

B220^+^IgM^-^IgD^-^ 5.5 ± 1.0 4.4 ± 1.7 5.9 ± 1.7 5.7 ± 1.8

B220^+^IgM^+^IgD^-^ 1.2 ± 0.5 1.0 ± 0.3 1.1 ± 0.4 0.9 ± 0.2

B220^+^ sIg^+^ 3.4 ± 1.1 1.6 ± 0.3*** 2.4 ± 1.2 1.3 ± 0.4***^

Mac1^+^Gr1^-^ 0.81 ± 0.17 1.3 ± 0.7* 0.85 ± 0.20 1.6 ± 0.9*^

Mac1^+^Gr1^+^ 9.3 ± 1.4 8.5 ± 2.4 10.1 ± 2.7 10.0 ± 3.3

**Thymus** 180 ± 49 239 ± 99 178 ± 39 240 ± 66*^

CD4^-^CD8^-^ 6.4 ± 3.0 6.1 ± 3.6 4.1 ± 1.0* 4.9 ± 1.5

CD4^+^CD8^+^ 155 ± 42 210 ± 86 154 ± 34 213 ± 54*^

CD4^+^CD8^-^ 12.4 ± 3.8 17.1 ± 9.1 14.5 ± 4.8 17.1 ± 8.8

CD4^-^CD8^+^ 6.6 ± 2.8 5.8 ± 2.7 5.9 ± 3.6 5.6 ± 3.5

Nucleated cells × 10^6^, except peripheral blood cells, × 10^6^/mL. Mean ± SD, 8-week-old male mice; n = 8-9 mice per genotype.

Student’s t-test:

*** Significantly different from WT (*P*≤0.001)

** Significantly different from WT (*P*≤ 0.01)

* Significantly different from WT (*P*≤ 0.05)

^^^ Significantly different *MYC10* vs *FoxO3^-/-^MYC10* (*P*≤ 0.001)

^^ Significantly different *MYC10* vs *FoxO3^-/-^MYC10* (*P*≤ 0.01)

^ Significantly different *MYC10* vs *FoxO3^-/-^MYC10* (*P*≤ 0.05)

🞟🞟 Significantly different *FoxO3^-/-^* vs *FoxO3^-/-^MYC10* (*P*≤ 0.01)

🞟 Significantly different *FoxO3^-/-^* vs *FoxO3^-/-^MYC10* (*P*≤ 0.05)

**Table S2. Haemopoietic composition of *FoxO3^-/-^*Eµ-*myc* mice**

**Organ/cell type WT *FoxO3^-/-^* Eµ-*myc* *FoxO3^-/-^*Eµ-*myc***

**Peripheral blood** 7.9 ± 1.5 10.8 ± 1.2*** 24.2 ± 3.7*** 35.6 ± 6.9***^^^🞟🞟🞟

CD4^+^CD8^-^ 1.8 ± 0.3 2.4 ± 0.4** 2.1 ± 0.8 2.6 ± 0.7**

CD4^-^CD8^+^ 1.1 ± 0.2 1.5 ± 0.2** 1.2 ± 0.4 1.5 ± 0.4

B220^+^IgM^-^IgD^-^ 0.37 ± 0.13 0.36 ± 0.09 10.9 ± 2.8*** 15.2 ± 4.5***^🞟🞟🞟

B220^+^ sIg^+^ 3.8 ± 1.1 4.3 ± 0.9 7.5 ± 0.7*** 9.4 ± 2.0***^🞟🞟🞟

Mac1^+^Gr1^-^ 0.44 ± 0.13 1.01 ± 0.43** 1.1 ± 0.3*** 2.6 ± 1.0***^^^🞟🞟

Mac1^+^Gr1^+^ 0.25 ± 0.11 0.85 ± 0.34*** 1.1 ± 0.6** 3.3 ± 1.3***^^^🞟🞟🞟

Ter119^+^ 1.5 ± 0.7 2.4 ± 0.5** 6.2 ± 3.8** 7.6 ± 4.6**🞟🞟

**Spleen** 194 ± 52 259 ± 23** 421 ± 60*** 571 ± 101***^^🞟🞟🞟

CD4^+^CD8^-^ 13.1 ± 3.3 18.2 ± 6.0 10.0 ± 1.8* 11.5 ± 2.6🞟

CD4^-^CD8^+^ 9.1 ± 2.0 12.3 ± 3.8 7.5 ± 1.8 8.0 ± 1.6🞟

B220^+^IgM^-^IgD^-^ 7.9 ± 2.8 11.1 ± 1.5* 140 ± 44*** 164 ± 52***🞟🞟🞟

B220^+^ sIg^+^ 79.0 ± 12.8 82.6 ± 17.0 86.6 ± 17.4 90.1 ± 17.2

Mac1^+^Gr1^-^ 4.7 ± 1.3 8.5 ± 2.5** 6.0 ± 2.5 10.8 ± 3.2***^^

Mac1^+^Gr1^+^ 5.5 ± 1.5 11.7 ± 3.2*** 9.8 ± 5.0* 17.9 ± 4.9***^^🞟🞟

Ter-119^+^ 71.8 ± 35.3 114 ± 26* 158 ± 27*** 253 ± 109***^🞟🞟

**LN** 29.3 ± 8.3 27.7 ± 4.7 44.6 ± 6.8*** 47.9 ± 9.1***🞟🞟🞟

CD4^+^CD8^-^ 10.9 ± 2.8 10.9 ± 1.7 15.5 ± 2.1** 15.6 ± 2.4**🞟🞟🞟

CD4^-^CD8^+^ 7.2 ± 2.2 7.6 ± 1.3 9.0 ± 1.2 9.3 ± 1.7🞟

B220^+^IgM^-^IgD^-^ 0.66 ± 0.28 0.60 ± 0.22 12.8 ± 5.3*** 14.2 ± 5.2***🞟🞟🞟

B220^+^ sIg^+^ 10.1 ± 3.5 7.8 ± 1.7 4.5 ± 0.9*** 5.0 ± 1.6**🞟🞟

**BM** 47.2 ± 5.0 49.7 ± 4.4 52.4 ± 8.1 52.2 ± 9.0

Ter-119^+^ 14.3 ± 2.1 16.7 ± 2.5 5.9 ± 1.7*** 8.7 ± 3.0***^🞟🞟🞟

Thy1^+^ 0.77 ± 0.27 0.58 ± 0.08 0.29 ± 0.16*** 0.41 ± 0.18**🞟

B220^+^IgM^-^IgD^-^ 13.1 ± 2.4 11.5 ± 1.6 33.6 ± 5.7*** 28.1 ± 4.9***^🞟🞟🞟

B220^+^IgM^+^IgD^-^ 2.9 ± 0.7 2.3 ± 1.0 1.8 ± 0.5** 1.1 ± 0.4***^^🞟🞟

B220^+^ sIg^+^ 3.6 ± 0.9 2.5 ± 0.4** 1.7 ± 0.5*** 1.2 ± 0.4***^🞟🞟🞟

Mac1^+^Gr1^-^ 1.1 ± 0.2 1.2 ± 0.4 0.94 ± 0.28 0.82 ± 0.33*🞟

Mac1^+^Gr1^+^ 13.3 ± 1.6 15.5 ± 2.1* 8.1 ± 2.1*** 10.4 ± 1.8**^🞟🞟🞟

**Thymus** 300 ± 22 376 ± 31*** 346 ± 38** 391 ± 58***

CD4^-^CD8^-^ 8.6 ± 1.5 10.8 ± 1.9* 13.8 ± 3.9** 26.6 ± 12.5**^🞟🞟

CD4^+^CD8^+^ 259 ± 20 329 ± 28*** 295 ± 34* 329 ± 58**

CD4^+^CD8^-^ 19.9 ± 2.0 23.3 ± 1.7** 22.4 ± 2.5* 23.2 ± 7.4

CD4^-^CD8^+^ 12.5 ± 4.2 12.0 ± 2.3 14.9 ± 3.1 11.0 ± 4.6

Nucleated cells × 10^6^, except peripheral blood cells, × 10^6^/mL. Mean ± SD, 4-week-old male mice; n = 8-9 mice per genotype.

Student’s t-test:

*** Significantly different from WT (*P*≤0.001)

** Significantly different from WT (*P*≤ 0.01)

* Significantly different from WT (*P*≤ 0.05)

^^^ Significantly different Eµ-*myc* vs *FoxO3^-/-^*Eµ-*myc* (*P*≤ 0.001)

^^ Significantly different Eµ-*myc* vs *FoxO3^-/-^*Eµ-*myc* (*P*≤ 0.01)

^ Significantly different Eµ-*myc* vs *FoxO3^-/-^*Eµ-*myc* (*P*≤ 0.05)

🞟🞟🞟 Significantly different *FoxO3^-/-^* vs *FoxO3^-/-^*Eµ-*myc* (*P*≤ 0.001)

🞟🞟 Significantly different *FoxO3^-/-^* vs *FoxO3^-/-^*Eµ-*myc* (*P*≤ 0.01)

🞟 Significantly different *FoxO3^-/-^* vs *FoxO3^-/-^*Eµ-*myc* (*P*≤ 0.05)
